# The Pathogenic *ADAMTSL2* D167N Variant Causes Geleophysic Dysplasia–Like Connective Tissue Changes in Mice

**DOI:** 10.1016/j.ajpath.2026.03.002

**Published:** 2026-03-19

**Authors:** Connie Lin, Divya I. Sivakumar, Ana D. Alcocer, Sophia T. Gavalas, Nandaraj Taye, Deborah E. Seifert, Zerina Balic, Timothy J. Mead, Dirk Hubmacher

**Affiliations:** ∗ Department of Pediatrics, Case Western Reserve University School of Medicine, Cleveland, Ohio; † Orthopedic Research Laboratories, Leni & Peter W. May Department of Orthopedics, Icahn School of Medicine at Mount Sinai, New York, New York; ‡ Division of Pediatric Cardiology, University Hospitals Rainbow Babies & Children's Hospital, Cleveland, Ohio; § Mindich Child Health and Development Institute, Icahn School of Medicine at Mount Sinai, New York, New York

## Abstract

Geleophysic dysplasia (GD) is caused by recessive mutations in *ADAMTSL2* (a disintegrin and metalloprotease with thrombospondin type I motifs-2; GD1), or dominant mutations in *FBN1* (GD2) or *LTBP3* (GD3). GD is characterized by severe short stature and other skeletal abnormalities, characteristic facial features, thick skin, and hypermuscular build. Life-threatening complications can arise from progressive heart valve disease and narrowing of the large airways, resulting in approximately 33% mortality before the age of 5 years. Despite high childhood mortality and significant morbidity, no disease-modifying treatments exist for GD. To model disease progression and enable efficacy testing of mechanism-based therapeutic approaches, a mouse model for severe GD1 was generated by introducing the patient-specific *ADAMTSL2* c.499G>A (p.D167N) mutation into the mouse *Adamtsl2* locus. Homozygous *Adamtsl2*^D167N/D167N^ (D167N) mice had reduced postnatal survival and developed short stature. Radiographs demonstrated significantly shortened hind limb and forelimb bones with delayed mineralization and abnormally shaped vertebrae. Histologic investigation revealed a shortened growth plate, suggesting abnormalities in chondrogenesis. Cardiac histomorphometry revealed dysplastic aortic heart valves, consistent with progressive heart valve disease observed in patients with GD1. In the lungs, bronchial obstruction was observed, as previously reported for global *Adamtsl2* knockout mice, likely resulting in occlusion of the affected airways. Thus, the ADAMTSL2 D167N mouse model recapitulates key clinical manifestations of patients with GD1.

Geleophysic dysplasia (GD) 1 [GD1; Online Mendelian Inheritance in Man (OMIM); *https://www.omim.org*, last accessed March 30, 2026; number 231050] is caused by recessive pathogenic variants in a disintegrin and metalloprotease with thrombospondin type I motifs-2 (*ADAMTSL2*) (approximately 50% of cases), which encodes a secreted, regulatory extracellular matrix (ECM) protein.^1^^,^^2^ GD can also be caused by dominant pathogenic variants in *FBN1* (GD2; OMIM number 614185; approximately 50% of cases) or latent transforming growth factor-β (TGF-β)–binding protein-3 (*LTBP3*; GD3; OMIM number 617809; <1% of cases), encoding the ECM proteins fibrillin-1 and LTBP3, respectively.^3^, ^4^, ^5^, ^6^ GD belongs to a group of syndromic skeletal dysplasias, the acromelic dysplasias, which also include Weill-Marchesani syndrome, acromicric dysplasia, and Myhre syndrome.^7^, ^8^, ^9^ Signs of GD become apparent in the first year of life and include severe short stature (–3 to –6 SDs), short fingers and toes (brachydactyly), joint contractures, thick skin, characteristic facial features (happy face), and a hypermuscular build. Complications from progressive heart valve disease and narrowing of the large airways cause mortality in approximately 33% of affected children before the age of 5 years.^10^ Despite high childhood mortality and lifelong morbidity, no disease-modifying treatments for GD1 are available. More recently, the neonatal lethal Al-Gazali skeletal dysplasia had been linked to recessive pathogenic variants in *ADAMTSL2*, likely representing the most severe end of the phenotypic spectrum of *ADAMTSL2*-related disorders.^11^ A dominant pathogenic variant in *ADAMTSL2* was also described, where patients presented with features opposite to the signs and symptoms of GD, including joint hypermobility and generalized tissue fragility of internal and external organs.^12^

ADAMTSL2 belongs to the seven-member ADAMTS-like family of regulatory ECM proteins.^13^ ADAMTS-like proteins share homology with the C-terminus of ADAMTS proteases but lack their protease domain. *ADAMTSL2* is expressed in several cell types and tissues, including bronchial smooth muscle cells, cardiac fibroblasts, tenocytes, skeletal muscle stem cells, the liver, and the brain vasculature.^2^^,^^14^, ^15^, ^16^, ^17^, ^18^, ^19^ In fibroblastic cell lineages, such as skin fibroblasts, cardiac fibroblasts, and fibroadipogenic progenitor cells in skeletal muscle, ADAMTSL2 is a negative regulator of TGF-β signaling.^1^^,^^19^, ^20^, ^21^ Mechanistically, ADAMTSL2 binds to fibrillin-1 and LTBP1, a key regulator of TGF-β signaling in the ECM, where it regulated the amount of LTBP1 that was incorporated into the ECM.^1^^,^^19^ As such, ADAMTSL2 deficiency in GD1 could result in elevated TGF-β signaling through altered latent TGF-β incorporation into the ECM or increased latent TGF-β activation. Challenging the involvement of elevated TGF-β signaling in the pathogenesis of GD1 are studies using mouse models and Al-Gazali skeletal dysplasia patient-derived fibroblasts, where TGF-β signaling was unchanged.^11^^,^^22^^,^^23^ In addition to regulating TGF-β signaling, ADAMTSL2 also regulates WNT signaling in the context of skeletal muscle stem cell differentiation.^15^ Here, ADAMTSL2 bound directly to canonical and noncanonical WNT ligands and components of the WNT receptor complex and promoted WNT signaling. Overexpression of ADAMTSL2 accelerated the differentiation of skeletal muscle stem cell–derived myoblasts into myotubes *in vitro* and accelerated skeletal muscle regeneration after injury *in vivo*.^15^^,^^24^

To further understand the pathogenesis of GD1 and to enable testing of candidate therapeutic approaches, a GD1 mouse model was developed and characterized by knocking-in the patient-specific *ADAMTSL2* c.499G>A (p.D167N) variant, which was described previously in an individual with severe GD1.^25^ This model recapitulated postnatal lethality, bone shortening, and bone shape changes as well as alterations in heart valves, similar to findings in patients with GD1.

## Materials and Methods

### Generation of *Adamtsl2*-D167N Mice

D167N mice were generated by clustered regularly interspaced short palindromic repeats (CRISPR)/CRISPR-associated protein 9 (Cas9)–mediated mutagenesis in C57Bl/6 mice in collaboration with Taconic/Cyagen (Santa Clara, CA). A DNA oligonucleotide (5′-GCCATGCGACCTACACTGCAGCACCGTGGACGGCCAACGGCAGCTCACGGTGCCGGCCCGTAACGGCACTTCCTGCAAGCTCACCGACCTGAGAGGGGTTTGCGTGTCTGGAAAATGTGAGGTT-3′) introducing the p.D167N (GAC to AAC) mutation and a synonymous CGA to CGT mutation (p.R166) was co-injected with Cas9 enzyme into fertilized mouse eggs to generate targeted knock-in offspring. The mutant codons are underlined in the primer sequence to indicate their location. The following guide RNAs were used (forward strand: 5′-CTCACGGTGCCGGCCCGAGACGG-3′; reverse strand: 5′-CAGGAAGTGCCGTCTCGGGCCGG-3′). The silent A>T variant was introduced to facilitate the identification of the mutant D167N allele by restriction digestion. F_0_ founder animals were identified by PCR followed by sequence analysis, which were subsequently bred to wild-type (WT) mice to achieve germline transmission and the generation of F_1_ mice. On import into the mouse facility at the Center for Comparative Medicine and Surgery at the Icahn School of Medicine at Mount Sinai (New York, NY), heterozygous D167N/+ mice were intercrossed to generate homozygous D167N mice and WT littermate controls for phenotypic analysis. Mice were housed in a temperature-controlled facility with *ad libitum* access to food and water and a 12-hour light/dark cycle. Before tissue harvest, mice were euthanized by CO_2_ asphyxiation, followed by cervical dislocation. All mouse experiments were approved by the Institutional Animal Care and Use Committee of the Icahn School of Medicine at Mount Sinai (protocol numbers PROTO202000259 and TR202300000105).

### Genotyping

Genomic DNA isolated from toes clipped for identification was used as a PCR template with the following primers: forward: 5′-CTTTCTTTGCAAGCCTCACTTTCT-3′; reverse: 5′-CTGTGATGGAGTACTCTTCTCCAC-3′. The following PCR conditions were used: initial denaturation at 94°C for 3 minutes, denaturation at 94°C for 30 seconds, annealing at 60°C for 30 seconds, extension at 65°C for 1 minute, repeat 33 times, and final extension at 65°C for 10 minutes. The PCR product was digested with BsoBI (New England Biolabs, Ipswich, MA), and fragments were separated with a 2% agarose gel containing ethidium bromide for visualization under ultraviolet light. The size of the band originating from the D167N allele was 424 bp. The sizes of the bands from the WT allele after BsoBI cleavage were 200 and 224 bp. The 100-bp DNA Ladder (New England Biolabs) was used as marker.

### ADAMTSL2 D167N Secretion Assay

The D167N (*GAA*>*AAC*) mutation (underlined, italics) was introduced in a previously published expression plasmid for C-terminally Myc/His-tagged full-length human ADAMTSL2 using BmgBI restriction and a BmgBI-restricted (sites underlined) gBlock (Integrated DNA Technology, Coralville, IA): 5′-CAGGACCTGCACGGGCACGTCCAAGCGGTACCAGCTCTGCAGAGTGCAGGAGTGTCCGCCGGACGGGAGGAGCTTCCGCGAGGAGCAGTGCGTCTCCTTCAACTCCCACGTGTACAACGGGCGGACGCACCAGTGGAAGCCTCTGTACCCGGATGACTATGTCCACATCTCCAGCAAACCGTGTGACCTGCACTGTACCACCGTGGACGGCCAGCGGCAGCTCATGGTCCCCGCCCGC*AAC*GGCACATCCTGCAAGCTCACTGACCTGCGAGGGGTTTGCGTGTCTGGAAAATGTGAGCCCATCGGCTGTGACGGGGTGCTTTTCTCCACCCACACACTGGACAAGTGTGGCATCTGCCAGGGGGACGGTAGCAGCTGCACCCACGTGACGGGCAACTATCGCAAGGGGAATGCCCACCTTGGTTACTCTCTGGTGACCCACATCCCGGCTGGTGCCCGAGACATCCAGATTGTAGAGAGGAAGAAGTCCGCTGACGTGCTAGCTCTTGCAGAT-3′ and T4 DNA Ligase (New England Biolabs).^16^ Plasmids were amplified in *Escherichia coli* DH5α and purified using standard molecular biology techniques. The sequence was verified using commercial Sanger DNA sequencing.

Human embryonic kidney (HEK) 293 cells (CRL-1573) were purchased from ATCC (Manassas, VA) and cultured in Dulbecco's modified Eagle’s medium supplemented with 10% fetal bovine serum, 1% l-glutamine, 100 units/mL penicillin, and 100 mg/mL streptomycin (complete Dulbecco's modified Eagle’s medium) in a 5% CO_2_ atmosphere in a humidified incubator at 37°C. For transient transfections, HEK-293 cells were seeded in 6-well cell culture plates and transiently transfected at 60% to 80% confluence with 1 μg of purified plasmid DNA using polyethylenimine reagent (Polysciences, Warrington, PA) in a 1:1 (w/w) ratio to plasmid DNA in serum-free Opti-minimal essential medium (Thermo Fisher Scientific, Waltham, MA). At 24 hours after transfection, HEK-293 cell layers were carefully rinsed with phosphate-buffered saline and cultured in serum-free Dulbecco's modified Eagle’s medium.

After 48 hours, conditioned medium was collected and cleared by centrifugation. The cell layer was rinsed with phosphate-buffered saline and lysed in lysis buffer (0.1% NP-40, 0.05% deoxycholate, and 0.01% SDS in phosphate-buffered saline). Equal volumes of conditioned medium and cell lysates were combined with 5× reducing SDS loading buffer, boiled at 100°C, and separated via SDS-PAGE for Western blot analysis. Proteins separated on polyacrylamide gels were blotted onto polyvinylidene difluoride membranes (Immobilon-FL; Merck Millipore, Burlington, MA) using a wet transfer system for 1.5 hours at 70 V at 4°C in 25 mmol/L Tris, 192 mmol/L glycine, and 20% methanol as transfer buffer. Membranes were blocked with 5% (w/v) milk in tris-buffered saline (TBS; 10 mmol/L Tris-HCl, pH 7.2, and 150 mmol/L NaCl) for 1 hour at room temperature and incubated with a monoclonal anti-Myc antibody diluted or a monoclonal antibody against glyceraldehyde-3-phosphate dehydrogenase (1:1000; MilliporeSigma, Burlington, MA) in 5% (w/v) milk in TBS + 0.1% Tween 20 at 4°C overnight. Membranes were then rinsed with TBS + 0.1% Tween 20 3 × 5 minutes at room temperature and incubated with IRDye-goat–anti-mouse secondary antibodies [1:10,000 in 5% (w/v); Jackson ImmunoResearch Laboratories, West Grove, PA] in TBS + 0.1% Tween 20 for 2 hours at room temperature. Membranes were then rinsed 3 × 5 minutes with TBS + 0.1% Tween 20, once in TBS, and imaged using an Azure c600 Western blot imaging system (Azure Biosystems, Dublin CA). Band intensities were quantified using the AzureSpot analysis software version 1.5.1105 (Azure Biosystems) and normalized to glyceraldehyde-3-phosphate dehydrogenase.

### X-Ray Imaging and Bone Length Measurements

Whole body and dissected limbs from sacrificed mice were imaged using a UltraFocus digital X-ray cabinet high-resolution radiographic system (Faxitron Bioptics, Hologic, Marlborough, MA). A 10-mm metal rod, imaged at the same magnification, was used as a scale to enable quantification of bone length. Images were exported in jpeg format and uploaded to ImageJ/Fiji version 1.52a (*https://fiji.sc*), where bone lengths were measured and absolute lengths were calculated on the basis of the length of the 10-mm metal rod.^26^

### Histology and Histomorphometric Measurements

All dissected tissue samples were fixed in 4% paraformaldehyde (Electron Microscopy Sciences, Hatfield, PA) prepared in phosphate-buffered saline at 4°C overnight, followed by paraffin embedding. Samples containing mineralized bone were first decalcified in 14% EDTA solution, with the decalcification medium changed every 3 days over a period of 3 to 4 weeks. Following deparaffinization and tissue rehydration, sections (7 μm thick) were prepared and mounted on SuperFrost Plus glass slides (Thermo Fisher Scientific). Sections were then stained with hematoxylin and eosin, Masson trichrome (AB150686; Abcam, Waltham, MA), safranin-O (350-16; MilliporeSigma), alcian blue 8GX (A3157; Sigma Aldrich, St. Louis, MO), and Movat pentachrome (KTRMPPT; StatLab, McKinney, TX) using experimental procedures, as previously described.^27^^,^^28^ Antigen retrieval was used prior to anti–Phospho-SMAD2 (1:200; #3108; Cell Signaling Technology, Danvers, MA) antibody staining by microwaving for 4 × 1.5 minutes in a microwave oven with 30-second intervals intervening prior to antibody incubation with citrate buffer [10 mmol/L sodium citrate, 2 mmol EDTA, 0.05% (v/v) Tween-20, pH 6.2]. Alexa Fluor 488–conjugated goat anti-rabbit immunoglobulin (1:400; A-11008; Molecular Probes, Invitrogen, Carlsbad, CA) was used as the secondary antibody and the nuclei were counterstained and mounted with Vectashield-DAPI mounting medium (Vector Labs, Burlingame, CA).

Heart valve thickness, length, and cross-sectional area was measured using ImageJ/Fiji version 1.52a.^26^ Valve thickness was determined by the widest portion of the cusps. Skin thickness and growth plate length were assessed by acquiring three independent measurements per section, with the mean value calculated for each sample. For airway analysis, three bronchi from each of the four lung lobes were examined per animal, totaling 70 bronchi across groups, and assessed for luminal occlusion.

Images were obtained using a Leica DM4 B microscope with K3M and K3C cameras and Leica LAS X software version 5.2.2 (Leica Microsystems, Boston. MA). Histologic sections were quantified using the ImageJ/Fiji software version 1.52a. Graphs were generated via Prism software version 10.5.0 (GraphPad Software, San Diego, CA), and images were generated using Adobe Photoshop Elements 2023 software (San Jose, CA).

### Statistical Analysis

Statistical analysis was performed with a two-tailed *t*-test for *n* = 2 samples or a one-way analysis of variance with a *post hoc* Tukey test for *n* > 2 samples. *P* < 0.05 was considered statistically significant. Calculations and graphing were performed with the OriginPro software version 10.2.0.188 (OriginLab, Northampton, MA) or GraphPad Prism software version 10.5.0. The sample size for each experiment is indicated in the respective figure legend.

## Results

### Generation of ADAMTSL2 D167N Mice and Molecular Consequences of the D167N Variant

**Figure 1:**
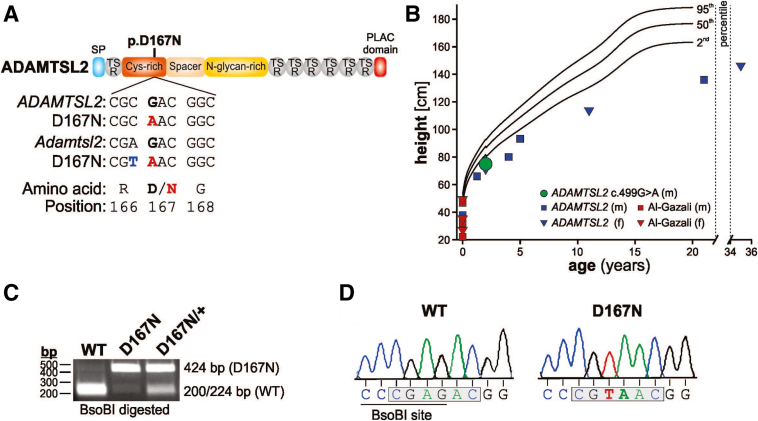
Generation of D167N mice. **A:** The *ADAMTSL2* c.499G>A (p.D167N) mutation (red nucleotides) was introduced into mouse *Adamtsl2*. The synonymous c.498A>T mutation (blue nucleotide) abolishes a BsoBI restriction site in the wild-type (WT) allele to facilitate the identification of mice harboring the mutant allele. **B:** Short stature in GD due to *ADAMTSL2* mutations. Each data point represents the reported height of a patient. The patient harboring the D167N variant (75 cm at 2 years of age) is depicted with a green circle. CDC growth curves for boys (95th, 50th, and 2nd percentile range) from 0 to 2 and 2 to 20 years are plotted as reference. **B:***x*-Axis break (**dashed lines**) was used. **C:** BsoBI restriction of an *Adamtsl2*-specific PCR product amplified from genomic DNA (424 bp) shows digestion of the band from the WT allele (200/224 bp) but not the D167N allele (424 bp). BsoBI restriction of a D167N/+ derived PCR product resulted in digested (WT) and undigested (D167N) bands. **D:** Sanger sequencing traces of undigested WT and D167N-derived PCR products show the presence of the G>A mutation. The BsoBI restriction site is underlined. **Gray boxed areas**: codons for R and D/N. F, female; m, male; PLAC, protease and lacunin; SP, signal peptide; TSR, thrombospondin type 1 repeat.

To gain insights into pathomechanisms and disease progression and to enable candidate therapeutic testing, a mouse model for GD1 was developed by introducing the pathogenic *ADAMTSL2* c.499G>A (p.D167N) variant into the mouse *Adamtsl2* locus using CRISPR/Cas9 gene editing (Figure 1A). The D167N mutation is located in a conserved region of the ADAMTSL2 cysteine-rich domain. Mice homozygous for the *Adamtsl2* c.499G>A variant will be referred to as D167N throughout the article. The D167N variant was previously identified by Ben-Salem et al^25^ in a patient with severe GD1. This patient presented with narrow airways at birth, followed by frequent chest infections requiring hospitalization and oxygenation. At 2 years of age, the patient had short stature (75 cm; less than the third percentile) similar to other individuals with reported GD1 or acromelic dysplasias due to different genetic etiologies (Figure 1B), brachydactyly, joint contractures, abnormal bone shapes, and severe pulmonary stenosis due to a dysplastic pulmonary valve, and was walking on tiptoes. The patient was homozygous for the *ADAMTSL2* c.499G>A (p.D167N) mutation, and both parents were carriers. A silent A>T variant, introduced to facilitate genotyping, rendered a PCR product amplified from genomic DNA of D167N mice resistant to BsoBI digestion, resulting in a single 424-bp band amplified from genomic DNA of D167N mice, whereas BsoBI digestion of the band amplified from genomic DNA from WT mice resulted in a 200-/224-bp double band that could not be resolved on a 2% agarose gel (Figure 1C). The correct DNA sequence was validated by Sanger sequencing of PCR products amplified from the genomic DNA isolated from WT and D167N mice (Figure 1D).

**Figure 2:**
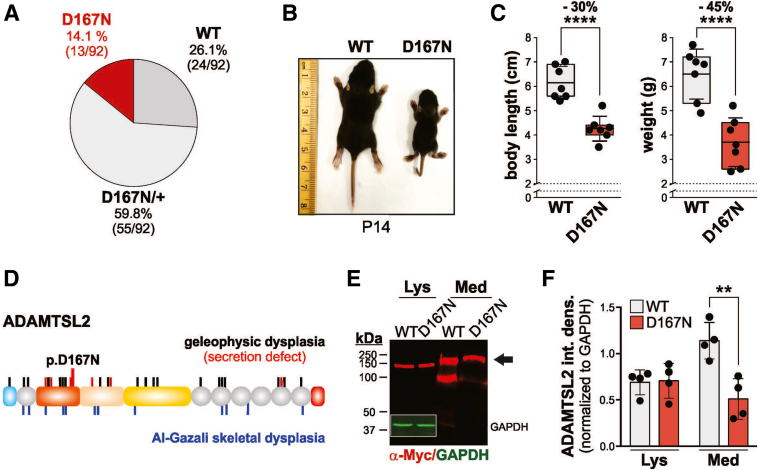
D167N mice develop short stature postnatally. **A:** Genotype distribution at postnatal day (P) 7 shows a reduced percentage of D167N pups (expected: 25%), indicating reduced survival. Numbers of pups/genotype are indicated. **B:** Appearance of wild-type (WT) and D167N mice at P14. **C:** Body length (tail to nose) and body weight of D167N mice at P14 are significantly reduced. *y*-Axis break (**dashed lines**) was used. **D:** Domain organization of ADAMTSL2 shows location of D167N and other GD1-causing mutations (**dashes**). Location of the ADAMTSL2 mutations that resulted in experimentally validated reduced protein secretion are indicated by **red dashes**. **E:** Western blot analysis detection of Myc-tagged WT and ADAMTSL2 D167N in cell lysate (Lys) or conditioned medium (Med) after transient overexpression in HEK-293 cells shows strong reduction of full-length D167N secretion. **Arrow** shows secreted full-length ADAMTSL2, and **boxed area** shows the bands representing glyceraldehyde-3-phosphate dehydrogenase (GAPDH) as loading control. **F:** Quantification of integrated density (int. dens.) of full-length ADAMTSL2 (upper band in medium samples) normalized to integrated density of GAPDH. **C** and **F:** Two-sample *t*-test was used. *n* = 92 mice (**A**); *n* = 7 mice per genotype (**C**); *n* = 4 (**F**). ∗∗*P* < 0.05, ∗∗∗∗*P* < 0.0001.

Heterozygous D167N/+ mice were viable and fertile as expected because of the recessive mode of inheritance of GD1-causing *ADAMTSL2* variants. At the time of genotyping between postnatal day (P) 7 and P10, the percentage of homozygous D167N mice was reduced (expected: 25%; observed: 14.1%), concomitant with an increase in the percentage of D167N/+ mice (expected: 50%; observed: 59.8%), suggesting some embryonic or early postnatal mortality (Figure 2A). In addition to reduced postnatal survival, D167N mice were smaller at P14, and their body length and body weight were significantly reduced (Figure 2, B and C). These data were consistent with postnatal mortality and short stature observed in patients with GD1 and conditional and global *Adamtsl2* knockout mice.^16^^,^^17^

To determine molecular consequences of the ADAMTSL2 D167N variant on protein secretion, the D167N mutation was introduced in an expression plasmid harboring C-terminally Myc/His-tagged ADAMTSL2 using site-directed mutagenesis (Figure 2D). ADAMTSL2 WT and D167N secretion was then quantified by Western blot analysis after transient transfection in HEK-293 cells (Figure 2, E and F). Secretion of full-length ADAMTSL2 D167N was strongly reduced by approximately 75% compared with full-length ADAMTSL2 WT, whereas no accumulation of ADAMTSL2 D167N in cell lysates was observed. This indicated that the D167N mutation impairs ADAMTSL2 secretion and, as such, behaves like other ADAMTSL2 mutations that had been previously analyzed in similar secretion assays.^20^^,^^29^^,^^30^ Because protease inhibitors were not used when collecting the conditioned medium, the possibility that the D167N mutation resulted in increased protease sensitivity, which could have contributed to the reduced amounts of ADAMTSL2 D167N, cannot be excluded. However, no apparent degradation products were observed on the Western blot analysis, except for the double band in secreted WT and D167N ADAMTSL2, which was observed previously and attributed to intramolecular proteolysis rather than differential glycosylation.^2^

### Bone Phenotypes in D167N Mice Are Characteristic of GD1

**Figure 3:**
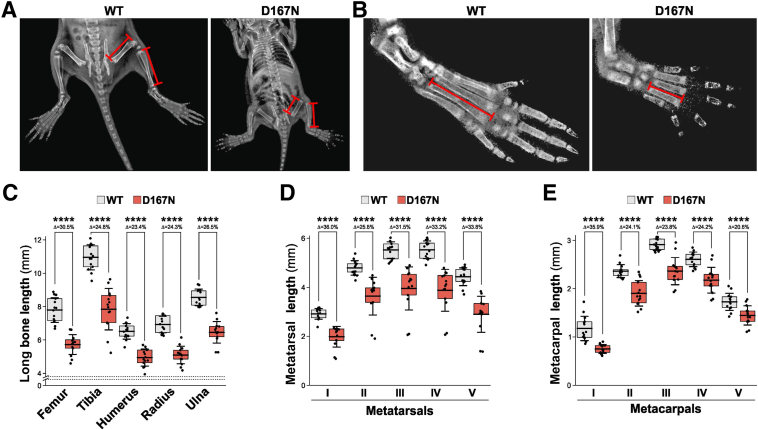
Bone shortening in D167N mice at postnatal day (P) 14. **A** and **B:** Radiographs of D167N mice at P14 show bone shortening (**A**), short, tubular metatarsals, and moth-eaten phalanges due to delayed bone age/mineralization (**B**). The **red brackets** indicate how the length of the skeletal elements was measured. **C**–**E:** Length measurements of long bones (**C**), metatarsals (**D**), and metacarpals (**E**) show significant bone shortening in D167N mice. **C:***y*-Axis break (**dashed lines**) was used. **C**–**E:** Two-sample *t*-test was used. *n* = 14 bones from 7 mice per genotype (**C**–**E**). ∗∗∗∗*P* < 0.0001. WT, wild type.

Because skeletal abnormalities, such as bone shortening, tubular bones, ovoid (egg-shaped) vertebral bodies, and delayed bone age/mineralization, are prominent features of GD1, bone lengths in WT and D167N mice at P14 were measured on the basis of X-ray images (Figure 3, A and B). D167N metatarsals and phalanges appeared moth eaten, indicating delayed mineralization (Figure 3B). Bone length measurements revealed significant shortening of long bones, metatarsals, and metacarpals (Figure 3, C–E). Because disproportional shortening of hands and feet is a feature of GD1 and other acromelic dysplasias, the average relative percentage of bone shortening in the D167N and WT forelimb (24.7%) and hind limb (27.6%) long bones and metacarpals (25.7%) and metatarsals (32.1%), respectively, were compared. However, statistically significant differences were not observed (*P* = 0.79 for forelimb long bones versus metacarpals; *P* = 0.23 for hind limb bones versus metatarsals). This suggested that at P14 the skeletal elements were shortened proportionally in D167N mice, and no distinct brachydactyly was observed.

**Figure 4:**
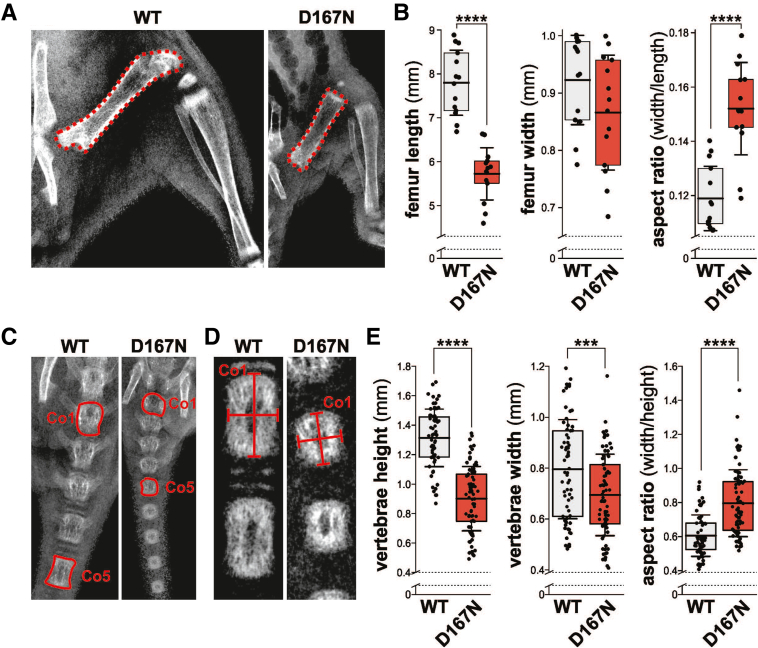
Bone shape changes in D167N long bones and vertebrae. **A:** Femur of wild-type (WT) and D167N mice. Bone shape is outlined with **red dashed line**. **B:** Quantification of D167N femur shape. Bone length (**left panel**), but not bone width at midshaft (**middle panel**), was significantly altered. **Right panel:** Aspect ratio (width/length) of D167N femurs was significantly increased. **C** and **D:** Low (**C**) and high (**D**) magnification shows altered D167N vertebrae shapes (outlined in red in **C** for orientation), reminiscent of ovoid vertebral bodies reported in patients with GD1. **D: Red lines** indicate the plane where the width and height of individual vertebrae was measured. **E:** Quantification shows decreased height (**left panel**) and width (**middle panel**) of D167N vertebrae, resulting in an increased aspect ratio (**right panel**). A total of 6 to 16 coccygeal vertebrae (Co) 7 mice per genotype were analyzed (total: 77 vertebrae per genotype). **B** and **E:***y*-Axis breaks (**dashed lines**) were used. **B** and **E:** Two-sample *t*-test was used. ∗∗∗*P* < 0.001, ∗∗∗∗*P* < 0.0001.

To analyze changes in bone shapes, the aspect ratio of the femur was determined. D167N femur length, but not its width, was significantly reduced, driving a significant increase in the femur aspect ratio (width/length), resulting in a tubular appearance (Figure 4, A and B). Similar changes were visually apparent in metatarsals (Figure 3B). Because ovoid vertebral bodies have been described in GD1, changes in vertebral shapes were examined by measuring the height and width of coccygeal vertebrae and calculating their aspect ratios (Figure 4, C–E). D167N vertebrae were less elongated and had a significantly reduced width (Figure 4E). This resulted in a significant increase in their aspect ratio consistent with an ovoid shape (Figure 4E). In Figure 4E, measurements for coccygeal vertebrae of different levels were combined.

Collectively, the D167N skeletal features were consistent with the bone phenotypes reported in patients with GD1.

### Skeletal Phenotypes Persist in D167N Mice at 6 Months of Age

**Figure 5:**
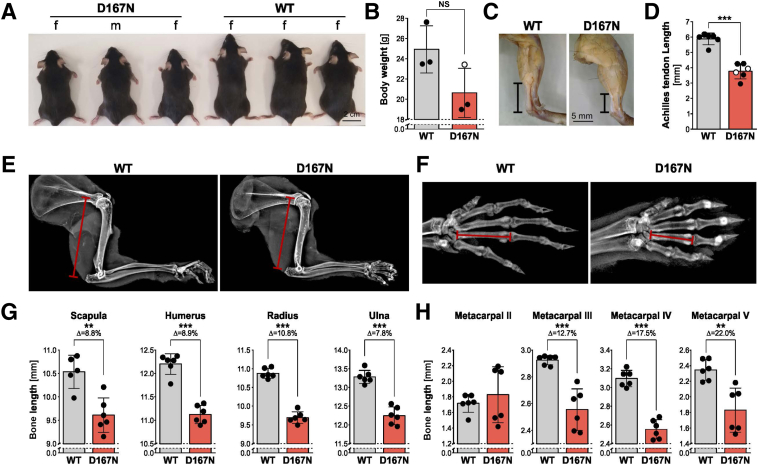
Gross and forelimb skeletal phenotypes of D167N mice at 6 months of age. **A:** Photographs showing appearance of D167N and wild-type (WT) littermates at 6 months of age. **B:** Body weight measurements comparing D167N and WT mice. Open circle: male; closed circles: females. **C:** Photograph of skinned hind limbs from D167N and WT mice (both female) with the Achilles tendon exposed. Its length is indicated with a **black bracket**. Note the apparently larger D167N hind limb muscles. **D:** Comparison of D167N with WT Achilles tendon length. Open circle: male; closed circles: females. **E** and **F:** Radiographs of 6-month–old forelimb (**E**) and hand (**F**) bones from D167N and WT mice. **Red lines** indicate length of humerus and metacarpal IV, respectively. **G** and **H:** Length measurements of forelimb long bones (**G**) and hand bones (**H**) from D167N and WT mice [six bones, except for scapula (five bones), from three mice per genotype]. **B**, **G**, and **H:***y*-Axis breaks (**dashed lines**) were used. **B**, **D**, **G**, and **H:** Two-sample *t*-test was used. *n* = 3 (**B**); *n* = 2 tendons from 3 mice per genotype (**D**). ∗∗*P* < 0.001, ∗∗∗*P* < 0.0001. Scale bar = 5 mm (**C**). F, female; NS, not significant; m, male.

Postnatal survival of D167N mice was variable, with some D167N surviving into adulthood. To determine if the skeletal phenotypes observed at P14 persisted, gross anatomic and bone phenotypes were analyzed in a cohort of littermates at 6 months of age. In whole-mount images, D167N mice appeared smaller compared with their littermates, which was supported by a trend toward a reduction in body weight (Figure 5, A and B). The reduction in body weight did not reach statistical significance because the only male in the D167N group had an increased body weight compared with the two D167N females. Because reduced Achilles tendon length in limb- and tendon-specific conditional *Adamtsl2* knockout mice was shown previously, Achilles tendon length in the WT and D167N mice was measured bilaterally.^15^ D167N Achilles tendons were significantly shorter compared with WT (Figure 5, C and D). In addition, D167N lower limb muscles appeared enlarged, which may be reflective of the pseudomuscular build described in patients with GD1 (Figure 5C). However, the weight of individual muscles was not determined.

**Figure 6:**
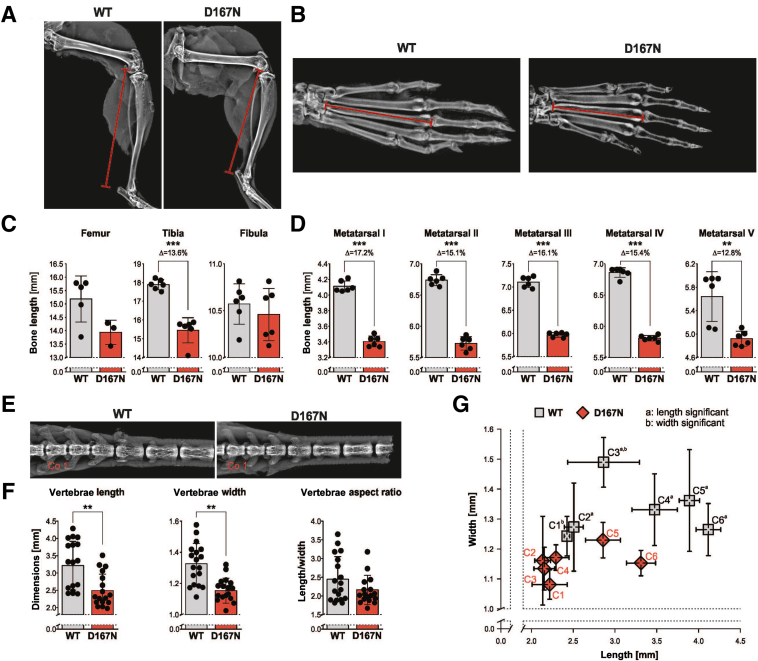
Hind limb and vertebral bone phenotypes of D167N mice at 6 months of age. **A** and **B:** Radiographs of 6-month–old hind limbs (**A**) and foot bones (**B**) from D167N and wild-type (WT) mice. **Red line** indicates length of tibia and metatarsal III, respectively. **C** and **D:** Length measurements of hind limb long bones (**C**) and metatarsals (**D**) from D167N and WT mice. **E:** Radiographs of coccygeal vertebrae (Co) from D167N and WT mice. Co 1 is indicated for orientation. **F:** Measurement of coccygeal vertebrae height (**left panel**) and width (**middle panel**) and calculated aspect ratio (**right panel**). Co 6 from 3 mice per genotype were analyzed (total: 18 vertebrae per genotype). **G:** Graphic representation of length and width of individual coccygeal vertebrae with SDs for length and width. **C**, **D**, **F**, and **G:***y*-Axis breaks (**dashed lines**) were used. **G:***x*-Axis break was used. **C**, **D**, **F**, and **G**: Two-sample *t*-test was used. *n* = 6 bones from 3 mice per genotype (**C** and **D**). ∗∗*P* < 0.001, ∗∗∗*P* < 0.0001.

Next, the lengths of individual forelimb bones were quantified on the basis of X-ray images (Figure 5, E–H). Like the P14 time point, the length of almost all 6-month D167N forelimb bones was significantly shorter compared with the respective WT bones. The only exception was metacarpal II, where the lengths were not significantly different between D167N and WT mice (Figure 5H). Similar differences were observed in hind limb bones, where all D167N bones, except the fibula, were shorter than the respective WT bones (Figure 6, A–D). In terms of the relative magnitude, an average of a 9.1% reduction in length of the D167N long bones in the forelimb and a reduction of 17.4% of the D167N metacarpals were observed (Figure 5, G and H). This difference was statistically significant (*P* = 0.017), suggesting disproportionally shorter hand bones, which would suggest brachydactyly, as described in patients with GD1 and other acromelic dysplasia. In the hind limbs, the difference in relative bone shortening between long bones (10.9%) and metatarsals (15.3%) showed a trend but did not reach statistical significance (*P* = 0.065) (Figure 6, C and D).

Finally, the aspect ratio of D167N and WT vertebrae at 6 months of age was determined by measuring length and width of coccygeal vertebrae (Figure 6, E–G). Both the length and the width of the D167N vertebrae were significantly shorter compared with WT vertebrae (Figure 6, F and G). Therefore, the aspect ratio was not different, indicating no changes in vertebra shape in D167N mice at 6 months of age.

### D167N Mice Have Reduced Bone Growth Plates

**Figure 7:**
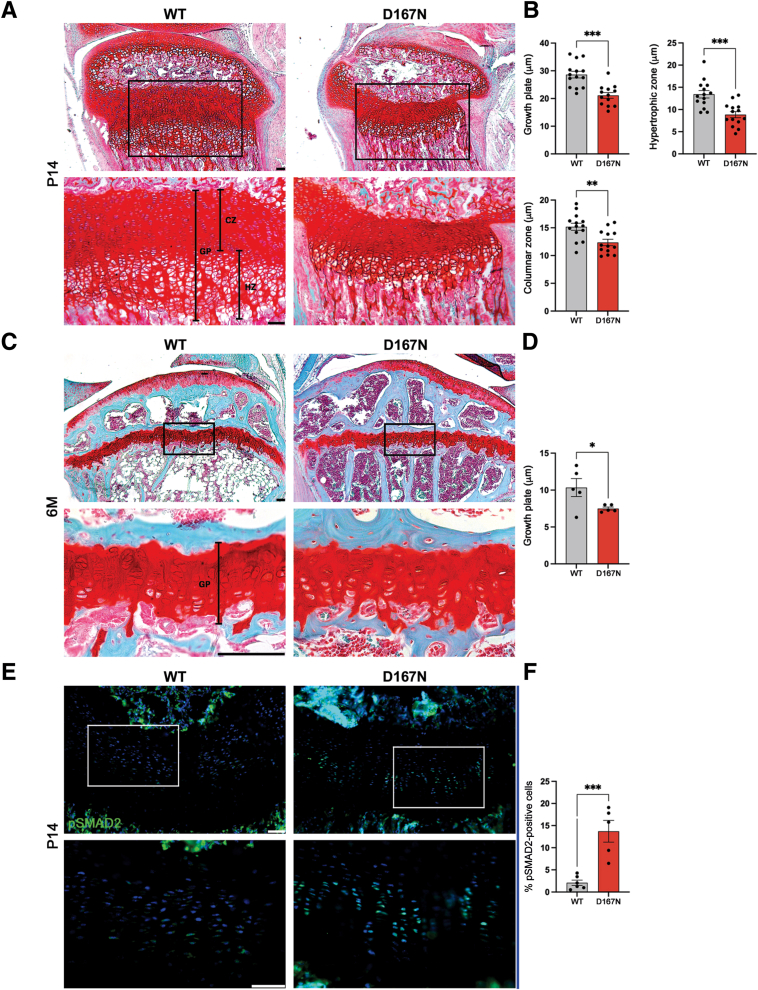
Shortened growth plate in D167N mice. **A:** Micrographs of safranin-O–stained proximal tibial growth plates (GPs) from postnatal day (P) 14 wild-type (WT) and D167N mice showing proliferative, columnar (CZ), and hypertrophic (HZ) zones. **B:** Quantification of the width of the growth plate and individual zones show reduced overall growth plate length, and reduced lengths of the columnar zone and hypertrophic zone at P14. **C:** Micrographs of safranin-O–stained proximal tibial growth plates from 6-month–old (6M) WT and D167N mice. **D:** Quantification of the width of the growth plate shows reduced growth plate length at 6 months of age in D167N compared with WT mice. **E:** Phosphorylated SMAD2 (pSMAD2) immunofluorescence staining of P14 WT and D167N GP chondrocytes. **F:** Quantification of percentage of pSMAD2-positive cells shows increased pSMAD2 positive staining in D167N GP chondrocytes compared with WT mice. **A**, **C**, and **E**: The **boxed areas** (**top panels**) depict the area magnified (**bottom panels**). **B**, **D**, and **F:** Two-sample *t*-test was used. *n* = 13 to 14 mice per genotype (**B** and **D**); *n* = 5 to 6 mice per genotype (**F**). ∗*P* < 0.05, ∗∗*P* < 0.01, and ∗∗∗*P* < 0.001. Scale bars: 100 μm (**A** and **C**); 50 μm (**E**).

Longitudinal bone growth is driven by the integrated processes of chondrocyte proliferation and maturation within the growth plate. Therefore, histologic analyses were conducted to examine growth plate architecture. Quantitative measurements on safranin-O–stained tibial sections showed a significant reduction in the total growth plate length in D167N mice compared with WT controls (Figure 7, A–D). Analysis of the distinct growth plate zones revealed a notable reduction in the height of both the proliferative (columnar) and hypertrophic zones in D167N mice at P14 compared with their WT counterparts (Figure 7, A and B). The columnar zone, responsible for chondrocyte division and longitudinal stacking, exhibited an average decrease of 18.6% in height. Similarly, the hypertrophic zone, where chondrocytes enlarge before apoptosis and ossification, was reduced by an average of 34.1%. As a result, the overall growth plate length shortened by an average of 26.2% in mutant mice.

To determine whether the observed abnormalities persisted in the mature skeleton, the proximal tibial growth plate from 6-month–old D167N mice was examined. Histologic examinations showed that the overall structure and zonal organization of the growth plate were comparable to those of age-matched WT controls (Figure 7C). However, consistent with the findings in D167N mice at P14, the growth plate (mainly hypertrophic zone) remained significantly reduced, with a 27.5% decrease in length compared with WT growth plates (Figure 7D).

Because ADAMTSL2 was previously implicated as a negative regulator of TGF-β signaling, P14 growth plates were immunostained for phosphorylated SMAD2, which is a key transducer of canonical TGF-β signaling (Figure 7, E and F). Quantitative histomorphometry revealed that the percentage of phosphorylated SMAD2–positive chondrocytes increased in D167N growth plates compared with WT controls at P14 (Figure 7F). This indicated that the phenotypic differences observed in D167N mice could result from overactivity of the TGF-β signaling pathway.

### Prominent Occlusions in D167N Mouse Lungs

**Figure 8:**
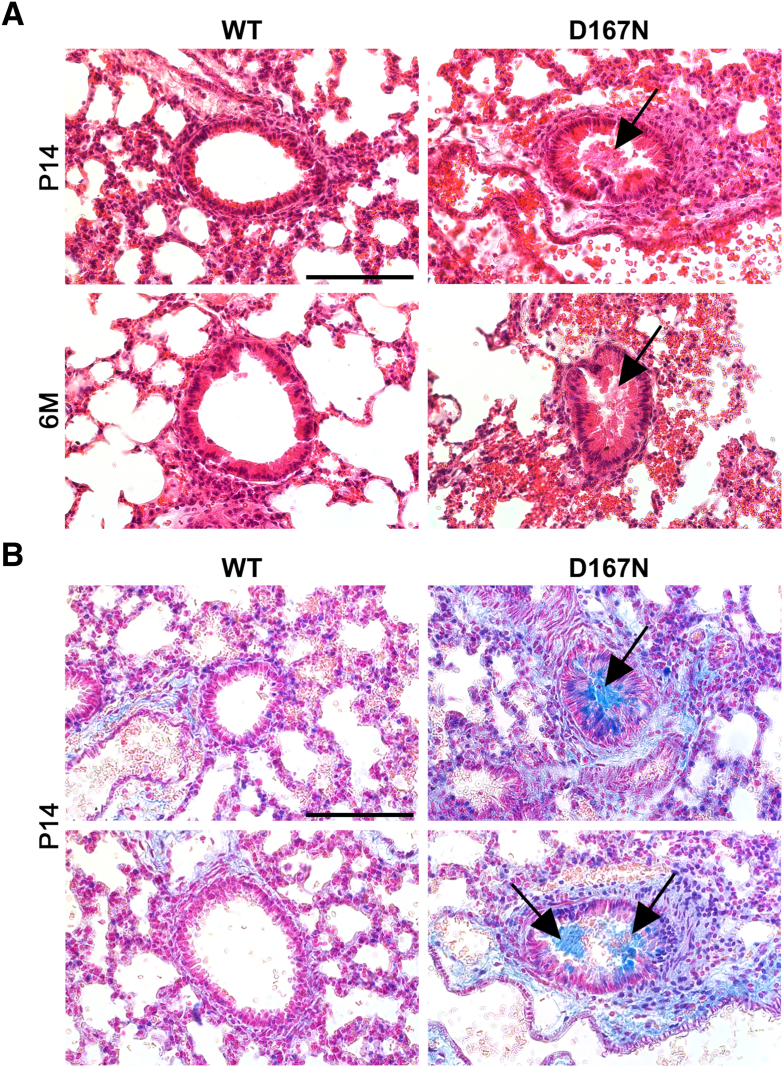
Bronchial occlusions in lungs from D167N mice. **A:** Hematoxylin and eosin–stained lung tissue from postnatal day (P) 14 and 6 months of age (6M) wild-type (WT) and D167N mice was imaged to visualize the bronchi. Eight bronchi were occluded with vesicle structures and dysplastic bronchial epithelium (**arrows**), whereas none was observed in WT lungs. **B:** P14 alcian blue–stained lung tissue showed positive staining in the vesicle structures in D167N bronchi (**arrows**). *n* = 5 P14 mice (**A**); *n* = 3 mice aged 6 months (**A**). Scale bar = 100 μm (**A** and **B**).

Pulmonary complications in GD1, including progressive airway narrowing and respiratory insufficiency, are a major cause of morbidity and mortality in patients with GD1. Histologic examination of lung sections from D167N mice identified focal, although infrequent, airway abnormalities, including dysplastic epithelium with luminal occlusion by vesicular structures (Figure 8A). A total of 8 of 70 examined bronchi displayed such features in D167N lungs, but none was observed in lungs from WT mice. Alcian blue staining identified the D167N bronchial occlusions as acidic mucin, which was consistent with previous findings (Figure 8B).

### Heart Valve Dysplasia in D167N Mice

**Figure 9:**
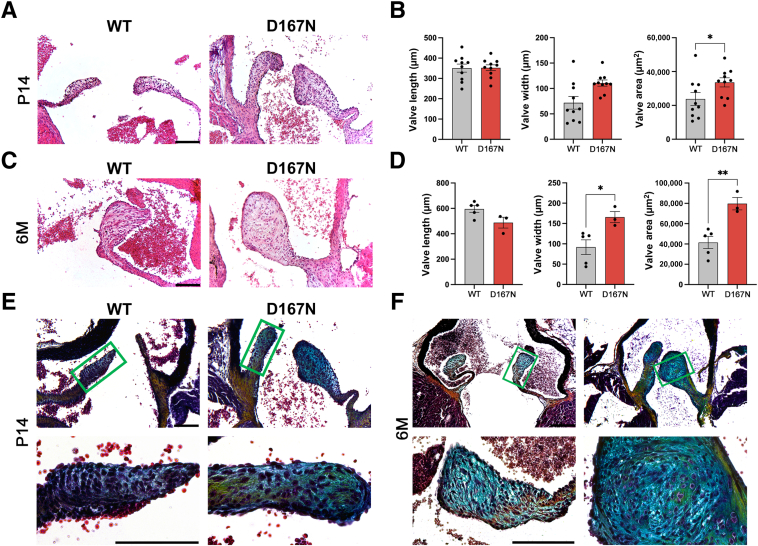
Increased aortic valve area in D167N mice. **A:** Micrographs of hematoxylin and eosin (H&E)–stained aortic valves from postnatal day (P) 14 wild-type (WT) and D167N mice showing dysmorphic leaflets in D167N mice. **B:** Quantification of average leaflet length, width, and area of P14 aortic valves from WT and D167N mice. **C:** Micrographs of H&E-stained aortic valves from 6-month–old (6M) WT and D167N mice. **D:** Quantification of average leaflet length, width, and area of aortic valves from 6-month–old WT and D167N mice. **E** and **F:** Aortic valves from P14 (**E**) and 6M (**F**) WT and D167N stained with pentachrome to visualize valve tissue architecture and composition. The **green boxed areas** depict zoomed-in images of the corresponding panels above. Blue, proteoglycans; yellow, collagen. **B** and **D:** Two-sample *t*-test was used. *n* = 10 mice per genotype (**B**); *n* = 5 WT mice (**D**); *n* = 3 D167N mice (**D**). ∗*P* < 0.05, ∗∗*P* < 0.01. Scale bar = 100 μm (**A**, **C**, **E**, and **F**).

Heart valve dysplasia is frequently observed in patients with GD1 and is associated with mortality. Morphometric analysis of aortic valve leaflets in D167N mice at P14 revealed a significant increase in leaflet area compared with WT controls (Figure 9, A and B). Aortic valve leaflet enlargement persisted with age, as 6-month D167N leaflets exhibited both increased area and width relative to age-matched WT valves (Figure 9, C and D).

**Supplemental Figure S1:**
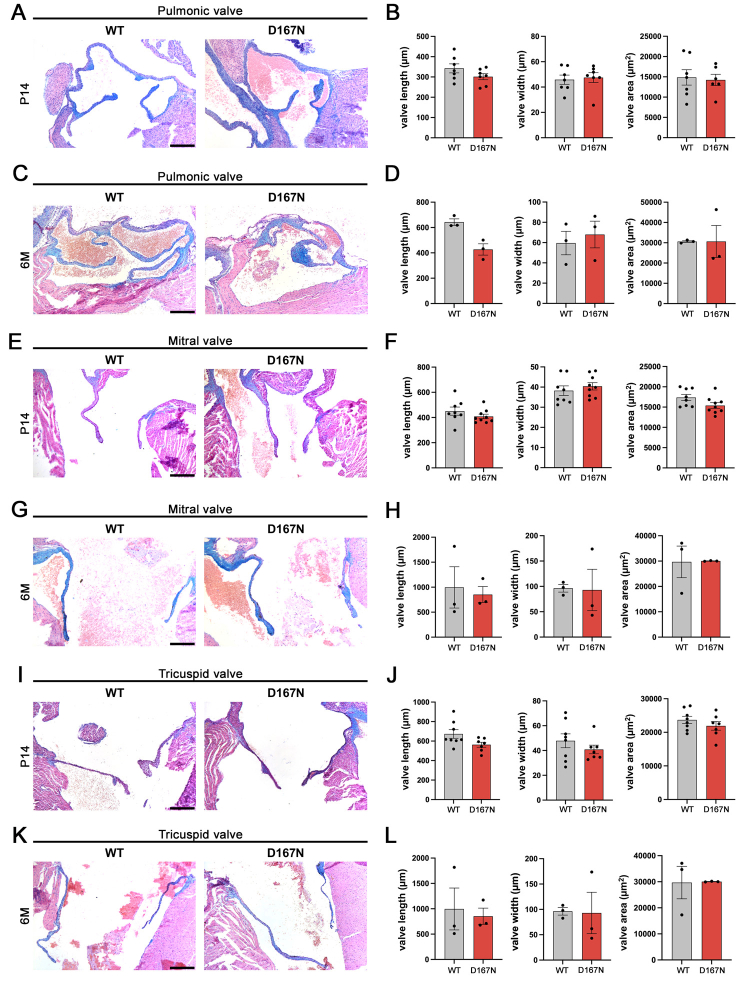
There are no histomorphometric changes in pulmonic, mitral, or tricuspid valves in D167N mice. **A**, **C**, **E**, **G**, **I**, and **K:** Micrographs of alcian blue–stained pulmonic, mitral, and tricuspid valves from postnatal day (P) 14 (**A**, **E**, and **I**) and 6 months of age (6M; **C**, **G**, and **K**) wild-type (WT) and D167N mice showing normal leaflets in D167N mice. **B**, **D**, **F**, **H**, **J**, and **L:** Quantification of average leaflet length, width, and area of P14 (**B**, **F**, and **J**) and 6M (**D**, **H**, and **L**) pulmonic, mitral, and tricuspid valves from WT and D167N mice. **A**, **D**, **E**, **H**, **I**, and **L:** Two-sample *t*-test was used. *n* ≤ 7 WT mice (**B**, **D**, **F**, **H**, **J**, and **L**); *n* = 10 D167N mice (**B**, **D**, **F**, **H**, **J**, and **L**). Scale bar = 100 μm (**A**, **C**, **E**, **G**, **I**, and **K**).

Histologic evaluation with Movat pentachrome staining highlighted pronounced differences in ECM composition and structure between mutant and control leaflets. Compared with WT valves, the D167N mutant leaflets exhibited disorganized and fragmented collagen fibers and an increase in proteoglycan content at both P14 and 6 months of age, indicative of ECM disorganization (Figure 9, E and F). Surprisingly, analysis of pulmonic, mitral, and tricuspid valve leaflets revealed no significant differences in leaflet area between D167N mutants and WT controls at either P14 or 6 months of age (Supplemental Figure S1).

## Discussion

Here, the phenotype of a novel, patient-specific mouse model for GD1 caused by mutations in *ADAMTSL2* was described. Homozygous D167N mice recapitulate cardinal cardiac and skeletal phenotypes that characterize patients with GD1, including heart valve dysplasia and bone shortening. In addition, evidence for reduced secretion or increased proteolytic susceptibility of the mutant ADAMTSL2 protein *in vitro* is provided, suggesting an underlying pathogenic mechanism that is in line with previous reports.^20^^,^^22^^,^^30^

A key sign of GD is short stature, which can be present already at birth or manifest during early postnatal growth, with a typical final height of –3 to –6 SDs.^10^ The bone and growth plate anomalies observed in the D167N GD1 mouse model are consistent with such short stature. Morphologically, a reduction in the length of most bones and associated changes in bone shapes resulted in more tubular bones and ovoid vertebral bodies. In the growth plate, a reduction in the width of the proliferative and hypertrophic zone resulted in an overall reduction in growth plate width. These findings would suggest a chondrocyte-regulatory function of ADAMTSL2, which is supported by a prior study from Delhon et al,^31^ where *Adamtsl2* was inactivated in the growth plate using *Col2a1*-Cre. In these mice, a reduction in the hypertrophic zone associated with enlarged chondrocytes was observed, suggesting a direct regulatory effect of ADAMTSL2 on chondrocyte proliferation and hypertrophic expansion. When comparing the growth plate anomalies of D167N mice with mouse models of other acromelic dysplasias, it is notable that they differ depending on the specific gene that was inactivated or mutated, even though they all converge in a short stature phenotype. For example, mice harboring a Weill-Marchesani syndrome patient-specific *Adamts10* knock-in mutation developed a shorter proliferative zone and an expanded hypertrophic zone, whereas global *Adamts10* knockout mice did not develop bone shortening.^32^^,^^33^ In an *Adamts17* knockout model, the width of the hypertrophic zone was reduced, but the proliferative zone was unaffected.^34^ In a acromelic dysplasia model due to a dominant fibrillin-1 (*Fbn1*) mutation, growth plate width was reduced and chondrocytes were disorganized.^35^ Together, these findings suggest that the genes mutated in acromelic dysplasias, including GD1, may affect different aspects of the regulation of chondrocyte and growth plate function with the same result (ie, short stature). Mechanistically, dysregulation of the bone morphogenetic protein (BMP) and TGF-β signaling pathways in the growth plate has been suggested, which could be related to dysregulated fibrillin-1 microfibril networks in the ECM.^31^^,^^32^^,^^34^^,^^36^ Indeed, increased activation of canonical TGF-β signaling in D167N growth plates was observed. However, TGF-β–inhibiting interventions would be required to determine if such elevated TGF-β signaling is disease-driving in GD1.

One of the clinically most consequential finding in patients with GD1 is heart valve dysplasia, which significantly contributes to morbidity and early mortality.^10^ In line with these clinical observations, early-onset and persistent enlargement of the aortic valve was observed, along with notable disorganization of the ECM in homozygous D167N mice. The combination of increased leaflet thickness and disrupted collagen deposition throughout the valve is characteristic of myxomatous degeneration, a pathologic process associated with functional impairment. These findings suggest that the D167N mutation induces early and progressive structural abnormalities in aortic valve architecture, leading to compromised tissue integrity and biomechanical function. This is consistent with GD1 case and autopsy reports showing aberrant function and shape of heart valves.^6^^,^^37^^,^^38^ In a separate study using a mouse model carrying the p.A165T ADAMTSL2 variant, cardiac anomalies, including impaired cardiac function, a narrowed aortic root, and enlarged cardiomyocytes, have been reported.^22^ In that model, unusual cell clusters with chondrocyte-like characteristics that could ultimately lead to ectopic cardiac valve calcification were shown in the atria and aortic valve by histology, but heart valve enlargement was not measured.^22^^,^^39^ In the D167N valves, such cell clusters were not observed and cardiac valve calcification was so far not described in patients with GD1. Interestingly, the pathologic changes in D167N mice were valve type specific. Although the aortic valve was clearly affected, no significant abnormalities were observed in the pulmonic valve at either developmental stage. In contrast to the D167N model, case reports for GD1 documented the variable involvement of all valve types in patients with GD1, including in the index patient harboring the D167N variant.^10^^,^^25^^,^^40^ This may suggest that the aortic valve is particularly vulnerable to disrupted ADAMTSL2 activity, at least in mice.

Other organ systems that are prominently affected in GD1 are the lung and skin. In lungs from D167N mice, noticeable bronchial occlusions were found, albeit in low frequency, which were completely absent in WT littermates. These findings are consistent with a previous report, where such occluded bronchi in *Adamtsl2* knockout mice likely caused perinatal respiratory failure and death.^16^ Although these findings were limited in prevalence, they mirror aspects of the airway obstruction and epithelial remodeling described in the heart and trachea from patients with GD1, suggesting that the D167N mutation may predispose to localized pulmonary pathology, in particular airway narrowing.^37^^,^^40^ Skin thickening is characteristic for acromelic dysplasias, including GD1.^10^ Although thickening of the skin has been described in mouse models of Weill-Marchesani syndrome and a dog model harboring an *ADAMTSL2* mutation, there was no clear evidence of thicker or fibrotic skin in D167N mice at 6 months of age (data not shown).^30^^,^^32^, ^33^, ^34^^,^^36^^,^^41^ Skeletal muscle architecture, where ADAMTSL2 regulates WNT and TGF-β signaling, was not analyzed.^15^^,^^21^

In summary, the successful development of a mouse model that recapitulates key manifestations of severe GD1 will allow researchers to delineate common and tissue-specific molecular mechanisms driving GD1 progression and to evaluate mechanism-based candidate therapeutic approaches to attenuate GD1 progression. As such, this model is complementary to the existing GD1 and acromelic dysplasia models and expected to contribute toward unraveling the ECM regulatory network that is underlying the musculoskeletal presentations connecting the acromelic dysplasia group.

## Disclosure Statement

None declared.
